# Abstract of the Proceedings of the Buffalo Medical Association

**Published:** 1861-08

**Authors:** J. F. Miner

**Affiliations:** Sec’y.


ART. II.—*Abstract of the Proceedings of the Buffalo Medical Association.*

TUESDAY EVENING, July 2, 1861.

The Association met at the usual hour.

Present—Dr. C. C. F. Gay, President, in the Chair; Drs. Treat, Rochester, Samo, Eastman, Cronyn and Miner.

The minutes of the last meeting were read and approved.

*Dr. Rochester*, in commenting upon a case reported by Dr. Dayton at the last meeting, said he had no doubt it was an example of Roseola Vaccina, its appearance about the 8th day after vaccination being about the time in which it is usually declared. He had himself witnessed several examples in his own patients, and although the affection is sufficiently distinctive, it had been mistaken by the individuals or their friends for Rubeola, Scarlatina and Variola.

Admission of members being in order, Drs. E. L. Bissell and Charles Mead were unanimously elected members upon compliance with the By-Laws.

*Dr. Rochester*, in opening the discussion on Erysipelas, stated, that he would not occupy any time in speaking of the early symptoms and various forms and phases and locations of the affection. He regarded it as a general disorder, usually of a low or asthenic tendency, having epidemic and endemic properties, and likewise communicable from individual to individual. It was also subject to atmospheric hygrometic and climatric influences, being more prevalent and more severe in very cold and very moist weather, and perhaps in very hot. Its relations to scarlatina and peritiontis, (especially puerperal,) were very marked—the three diseases often prevailing in an epidemic form, simultaneously, and many a case of peritionitis could be traced distinctly to an erysipelatous exposure. This is chiefly true, as respects the puerperal condition, but instances are on record where menstruation has seemed to afford a way of communication ; cases illustrative of these positions, viz : epidemic and endemic influences ; communicability, and puerpero-peritoneal susceptibility, were then cited. As regards treatment, it is usually divided into general and local. Many cases required little of either ; those however that could properly be termed grave, generally required tonics, stimulants and nutritious diet. In former years, Dr. R. had seen persons bled and treated with tartar emetic, with apparent advantage, he now treated the same class of patients with beef essence, with at least as good results. He regarded opium in a full dose as extremely useful, where there was either delirium or great restlessness. Salines, either as cathartics or diuretics, are advantageous, but should not be administered very freely. Quinine and iron, one or both, are required in a majority of instances. The tinct. of the chloride of iron is a good form, and is thought by many to exercise specific control over the disease in question. Dr. Rochester thought both these agents excellent tonics, and by virtue of that property only, to act favorably.

As to *local* treatment, it often seemed to arrest the progress of the affection, while it also often was apparently useless, and sometimes even hurtful. A great variety of topical agents were used, a strong presumption against the special efficacy of any. Of the supposed line of demarkation, effected by the solid nitrate of silver, he spoke only to condemn it. The tinct. of iodine was useful, but probably inferior to the tinct. of the chloride of iron as a diffuse application.

The latter he believed was first employed in Buffalo as a topical remedy. A patient of Prof. White's, for whom it had been prescribed, used it, by mistake, as an external dressing, so good a result followed, that the case was reported, in connection with others also thus treated, by Prof. White. His experience was subsequently corroborated by that of other members of the Association, and, as has before happened, a fortunate mistake has been turned to good account. The action of the tincture is not unlike that of Velpeau's solution of sulphate of iron, and has the advantage of greater facility and neatness of application.

Evaporating, alcoholic, astringent, and anodyne lotions, all have their advocates. Prof. Hamilton almost habitually employs warm water on soft cloths. Prof. Henry G. Cox, of the Emigrants' Hospital, Ward's Island, N. Y., speaks very highly of dry carded cotton, especially where large portions of the trunk or limbs are involved.

Dr. Rochester concluded his remarks by reporting the following unique and fatal case of the disease under consideration :

Late in March, 1861, he was consulted by a private patient, who had long suffered from gleet ; he was thirty-six years old, of naturally good constitution, did not drink spirits, and was addicted to but one excess, that of venery. For the past year he had indulged in extreme and illicit sexual intercourse. He stated that for two days he had suffered with chills, nausea, and great prostration, and that one of his testicles was swollen and tender. Examination disclosed epididymitis, with unusual fullness and tenderness of the vessels of the cord. He was induced to take a private room in the Buffalo General Hospital, where it was hoped that with quietude, the supine position, and mild treatment, he would soon be relieved. At the end of three days there was no improvement, and the cord increasing in tenderness, two fresh, lively leeches, were applied over the cord as it emerges from the pubis, and a warm poultice directed to follow. One of the leeches was *torn out* roughly by the patient, leaving an irregular jagged wound. In six hours this wound began to discharge a purulent ichor, and was surrounded by a livid erysipelatous margin. This spread rapidly over the hip and thigh, and in twenty-four hours a gangrenous slough appeared near the middle of the hip. Deep and free incisions disclosed complete disintegration and puruloid infiltration of the muscular tissues. In spite of every effort at arrest, aided by the counsels of Drs. Miner and Wilcox, the *death* of the parts progressed, more gangrenous spots appeared, and the erysipelatous blush extended down the leg and over the abdomen. The patient died on the third day, after the application of the leeches. Did the leech bite originate the malignant and fatal erysipelas? or was the original disease phlebitis of the veins of the cord. Post-mortem examination revealed the femoral vein occluded by a coagulum of aplastic lymph.

*Prof. Eastman* remarked that he did not know the subject was to come up for discussion this evening, supposing it had been disposed of on a previous occasion. He had been very much interested in the remarks made by Prof. Rochester—and as allusion had been made to traumatic erysipelas—as it was developed in the wards of the Buffalo Hospital of the Sisters of Charity during the period of his attendance on the Surgical Wards of that Institution, he would give some account of the same.

Early in December last, a case of erysipelas appeared in the medical ward under the care of Prof. Rochester—occurring in a patient who had been an inmate of the Hospital for eighteen months. Four cases occurred in that ward in the course of six or eight weeks. Information of the existence of the disease in the medical ward was immediately given by Prof. R., and precautionary measures employed, in the hope of preventing its development in the surgical wards.

On the 5th December, 1860, M—— M——, aged 15, of strumous constitution, and thin, spare habit, was admitted with Iliac abscess. It was opened by a valvular incision, and a small amount of pus appeared, which continued to discharge, (increasing in amount,) from day to day. On the 21st December erysipelatous inflammation set in on the affected limb, and extended over the whole thigh. It was attended with the usual constitutional symptoms, and it did not abate, death occurring the fourth day after. No autopsy permitted.

Two or three days after, the ear of a patient in the Eye Ward was attacked with erysipelas. This ward is separated from the general surgical ward, by a wide wall. The constitutional symptoms were not severe. The disease doubtless arose from a blistered surface behind and adjoining the ear, and extended into the scalp and neck.

The next case occurred in the Female Ward, which is on the floor below the male eye ward, in the person of a patient who had been severely burned on her left shoulder, breast and arm. She had been in the hospital for six weeks before she was attacked with erysipelas. It involved the left forearm and side, and death ensued in about a week.

On the 14th January, 1861, a patient in the Male Ward, who had been an inmate of the Hospital for several months, with a chronic, indolent ulcer, was attacked with chills. On making my next visit, erysipelatous inflammation was observed, with diffused redness following the course of the veins of the same leg affected with the ulcer. The foot and limb were greatly swollen. The disease subsided, with a copious discharge of purulent matter from the ulcer.

On the 8th February, a patient was received from a distant city, who had come to the Hospital for the purpose of having his leg amputated. His general health was good, though some months since he had been quite intemperate. The amputation was made on the 13th by Prof Moore, in the presence of the medical class, at the amphitheatre of the Hospital, with but little loss of blood. In the afternoon of the same day, he had a severe chill, with nausea and vomiting. The next day erysipelas attacked the stump, and extended above the knee, the thigh becoming greatly swollen, and the track of the superficial veins red and tender on pressure. Vomiting continued incessantly, the stomach rejecting every thing taken into it. Neither were nutritive enemas retained. No union of the flaps occurred, and only a thin, sanious discharge, (small in amount,) exuded from the stump. Death ensued on the evening of the 17th. No autopsy.

About the same time another patient with a varicose ulcer had chills, followed in a day or two with the development of a diffused erysipelatous inflammation along the course of the veins of the affected limb, attended with marked tenderness on the slightest pressure. The disease terminated in resolution in the course of four or five days; from which time the ulcer healed rapidly, and the patient left the Hospital in better health than he had enjoyed for a long time.

On the 26th of February, M—— O'L——, was admitted to the Hospital, with a fracture of the lower end of the radius, which occurred on the 22d February. A wound of an inch in length existed on the ulna side of the fore-arm, near the ulno-carpal articulation which had been closed with a suture and straps. On the 27th he reported comfortable, and said his arm did not pain him. On the 28th, his arm was enormously swollen and universally reddened. The constitutional symptoms were severe. The splints had been removed, and a large poultice applied. On the 5th and 6th March, pus discharged freely from the wound on the ulna side of the fore-arm, and from that time the swelling diminished, though the pu continued to flow during the time he remained under my care, my term of service expiring on the first of April. The ulna and some of the carpal bones became carious, and in due time amputation of the arm was advised to which he would not consent. He has since left the Hospital, having lost one or two of the carpal bones, and the lower third of the ulna.

The sustaining treatment was employed in all of these cases. Tonics, (quinine and iron,) were freely used with concentrated nutriment; whiskey being added with anodynes, as necessity required. As a local application, much benefit was derived from the tinctura ferri chloride, applied with a camel's-hair pencil three or four times daily. It was also administered internally with good results.

During the period of my attendance upon the ward, hygienic measures were continued to prevent the spread or communication of the disease, but with little, or no avail. The patients were removed into a ward remote from the "infected district," and the general ward was thoroughly cleansed, fumigated and whitewashed. At the end of the week, the patients were taken back to the ward without my knowledge, after which the disease again appeared with as much malignity as ever.

*Dr. Cronyn* thinks almost all cases of traumatic erysipelas a wound or scratch upon the surface will have much to do, acting as an exciting cause. Related a case recently treated of injury followed by erysipelatous inflammation, and diphtheritic exudation extending over the shoulder and fore arm, with very great swelling, high fever, &c. Has used more recently mur. tinc. ferri.; Velpeau's solution has also used for years; has not any confidence in nit. silver as an application in erysipelas. Dr. C. relates a case of crushed finger, which he wanted to remove immediately—the patient objecting; three days later the finger was gangrenous, and erysipelatous inflammation extended over the whole hand; removed the finger, and patient recovered under the use of tonics and stimulants. Dr. Cronyn also spoke somewhat at length of the nature of idiopathic erysipelas. Has seen the disease treated by bleeding, but since the great change in medical practice, hardly thinks any intelligent physician would adopt it. Spoke of the distinctions made of erysipelas by English surgeons, and the common practice of calling almost every superficial redness, erysipelas, erythema being often called erysipelas, not only by the public, but also by physicians. Dr. C. prefers iron in erysipelas to quinine. Many high authorities in Europe do not regard quinine as a tonic.

*Prof. Rochester* thought quinine a tonic, and, according to its dose, both tonic and sedative, but not a stimulant.

*Dr. Samo* related a case of phlegmonous erysipelas, affecting the scrotum, producing extensive slough, and attended with great difficulty in healing, erections often tearing up the parts, and preventing union.

Agreed perfectly with what had been said as to the general principles of treatment.

*Dr. Treat* looks differently from most, or all others, upon this affection. In the neurotic form of erysipelas nit. silver acts favorably; tinc. of iron often useful, and also quinine; while in other forms of the disease we bleed, and give bitartrate potassa. Dr. T. also spoke of the neurotic character of other eruptive diseases, as illustrating his views upon the different forms in which erysipelas made its appearance.

*Dr. Miner* gave some account of erysipelas in the Buffalo General Hospital, at the same time when it was so common, troublesome, and fatal at the Hospital of Sisters of Charity. Several cases occurred in the wards, and no attempt was made to separate cases of erysipelas form others. The Hospital dresser had quite a severe attack, and was treated as a private patient, in his own room adjoining the ward. Flannel, wrung from quite warm water, gave most relief to the pain as a local application. Quinine and opium were relied upon as the chief remedies. Other cases not so severe appeared, and one patient in Surgical ward, in the last stage of disease, having extensive caries of spine was attacked, and died with it, and his former disease. What seemed remarkable was, though erysipelas was common in the ward, in rather mild form, yet no single case occurred after operation or injury, and though no operation could be made at the Hospital of Sisters of Charity, a short distance from us, with any degree of safety, yet we were altogether exempt from the disease in the traumatic form.

*Prof. Eastman* thinks that there must have been phlebitis before the leeches were applied in Dr. Rochester's ease, occurring in Buffalo General Hospital, from the very early suppuration in the place of the bite. Spoke of the great value of quinine in small doses of one or two grs., and also of its unquestionable value as a tonic.

*Dr. Treat* regards it as the most valuable tonic, and also claims very many other virtues as belonging to it.

*Dr. Gay* remarked that he had been interested and edified in listening to the discussion, and fully concurred in all that had been said, both in regard to the pathology and treatment of Erysipelas. He had seen the disease in different sections of the country. At Philadelphia, during the year 1848, no less a man than the late Dr. Mitchell, resorted to depletion, but, as he believed at the time, with bad results. When the disease occurred in the intemperate, both Dr. Mitchell and Dr. Wood prescribed alcoholic stimulants with a much better result. He had also seen the disease in the country, and believed that as its subjects were more vigorous and healthful, tonics were not so beneficial, convalescence being more complete and satisfactory without than with them; he must, therefore, again insist upon the supreme efficacy of his time-honored remedy, viz: calchicum, combined with nitrate potass, or pulv. Ipecacuanhæ composetus, as most appropriate for rural practice.

Dr. G. had also witnessed the progress of the disease in this City, both in private and hospital practice, and fully and freely acquiesces in the remarks made by Drs. Rochester, Eastman and others. Here tonics and stimulants are to be relied upon as a rule, while at the same time he would not ignore the fact, that there may be exceptions to the rule, requiring discrimination on the part of the physician in the adaptation of remedies in each particular case.

*Dr. Gay* also remarked that he thought in many instances the local affection was often regarded as the primary disease, and treated as *the* disease, loosing sight of the fact that it was a general disease; hence he believes much mischief had often ensued by the application of too powerful remedies locally. All that can safely be attempted with local application, is so to apply them as to modify the intensity of the local inflammation; anything beyond this he regards as hazardous.

The details of a case by Dr. Samo, calls to mind a case of St. Anthony's fire, when almost the entire surface of the body was covered with the rash, which remained out for twenty-four hours, and then apparently accumulating force by concentration, located upon the scrotum, which became enormously swollen, and in a few days gangreneous, resulting in death. The topical application in this case was Velpeau's solution of sulph. of iron. The general treatment quinine, mur. tinct. ferri. and stimulants. Though he regards St. Anthony's fire and erysipelas as synonymous, he had made use of the term for the reason it had usually been applied whenever the entire surface was affected, as in this case, at its inception. It is the part of wisdom in the treatment of this affection, as in all others, to bestow more thought upon the patient, and less upon the malady, adapting the means of relief to the particular case in hand.

*Dr. Treat* wants to know how they treat burns in the Hospital; what external applications are found best. Has two children under treatment for very bad superficial burns, with great shock; one is not likely to recover unless some counter shock can be made. Some surgeons are talking of turpentine as a very valuable application. Dr. T. does not like to use remedies with which he has no personal experience, unless well satisfied that no harm will result from it.

*Dr. Miner* speaks of the general principles of treatment in cases of large, superficial burns, the main object to be accomplished, being to protect the exposed nervous tissue from the irritation of the atmosphere and other influences, to form as nearly as could be an artificial skin. For this purpose various applications have been highly recommended. Oil and lime water are most commonly employed, being poured upon cotton and allowed to remain upon the exposed surface for several days, until pus covers the granulations, making nature's protection, and allowing the cotton and oil to be easily removed.

*Prof. Eastman* agreed with Dr. Miner as to the principles involved and the indications to be answered, and speaks favorably of the oil and lime water.

*Dr. Cronyn* had tried the turpentine plan of treatment, both arms being badly burned, he had poured upon one, turpentine and lard, the other, oil and chalk, carefully enveloping each arm in cotton. The one treated with turpentine recovered much the soonest.

*Dr. Gay* asked the intelligence of the Association to report a case of tuberculosis, and as the evening was considerably advanced, he would be as brief as possible. The general symptoms and physical signs at first were so obscure, the inception of the disease so insidious, and progress so rapid, terminating fatally the fifth week, as to furnish perhaps some points of interest.

*History.*—F—— H——, female, aged 16 years, previous health good, hereditary predisposition not ascertained.

November, 1860, she was treated for three weeks homœopathically for a chronic catarrh; at the expiration of which time I was called; her case at this time causing considerable alarm; found her anasarcus pulse 112 per minute, tongue red, stomach irritable, tenderness at epigastrum, catamenia retained, no albuminuria; conceiving it to be case of arsenical poisoning, prescribed syr. hypophosphate of ferri, one oz. three times daily; under this treatment the dropsy subsided, and she was convalescent in three weeks, but was left however with some pain occurring spasmodically on right side the chest, shooting up to right shoulder, which seemed to be relieved by the use of Fl. ext. belladonna.

January 28th.—Was called to attend her for rubeola, which ran its usual time, and from which she kindly convalesced. About the first of

March she accompanied her parents to New York City, spending a number of weeks. A few days after her return to Buffalo she had a cough. I prescribed a simple cough mixture without any relief.

May 9th.—Symptoms, pulse 120 per minute, respiration hurried when exercising, normal when at rest, cough at night, sometimes followed with vomiting, no expectoration, some complaint of pain in right side chest, bowels regular, no appetite, general debility, had not menstruated for two months. Exploration of chest revealed no signs of deposit, percussion and auscultatory sounds normal, no depression. ℞ pyrophosphate ferri gr. ij. ter. die. syr. Ipecac P. R. N., Croton and Olive Oil equal parts, to be rubbed on chest, generous diet and out-door passive exercise; the latter was, however, precluded by inclement weather; the uvula being elongated was exercised.

18th.—Cough better, scarcely any pain, no appetite, and no stronger; had ridden a short distance for two or three mornings. Increase iron grs. iv. ter. die. with whiskey and beef essence. Pulv. opii comp. at night.

21st.—Appears improved, pulse still frequent, appetite improved, is loosing flesh, physical signs negative.

25th.—Not so well, remains in bed, increase whiskey and beef essence, discontinue iron.

28th.—Marked depression at the infra-clavicular space on right side and dullness on percussion, with usual auscultatory signs of tuberculosis. Continue whiskey and ordered oleum morrhuæ.

June 6th.—After my morning visit, the patient was attacked with pain in stomach, followed with vomiting, continued to vomit during the day, ejecting large amount of fluid of a deep green color. Champaign was ordered which at once arrested the vomiting, she now had pain in the bowels, and two or three dejections.

7th.—Had a restless night, no more vomiting nor diarrhœa, no cough nor pain, comfortable and cheerful ; very great depression beneath right clavicle and great dullness. 6 o'clock P. M.,—had a comfortable day, taken considerable nourishment and stimulus.

8th, 6 o'clock A. M.—Was summoned to her bed-side in haste ; has had another restless night ; pain now severe in bowels, extremities cold, lips livid, pulseless at wrist, died in an hour and a half.

*Remarks.*—The only general symptom of tuberculosis was the frequency of the pulse, which in the absence of fever or local inflammation, lead me to appreciate the nature of the case. The physical signs for the first three weeks contributed no assistance toward forming a correct diagnosis. At the end of the third week the physical signs were unmistakable. There was no softening, and there was no reason to believe my patient might not live for weeks, or even months, had not the vomiting supervened. But I cannot think she died from exhaustion, from the fact of her comfortable state the day prior to her death. The vomiting occurred at the time of her accustomed period for menstruation. Had there been no vomiting, there doubtless would have been an exhausting diarrhœa.

The rapid death was in keeping with the rapid formation and accumulation at the apex of the right lung of the tuberculous deposit. The disease no doubt may be dated back to her former sickness, or perhaps was antecedent to it, the deposit being so minute and diffused as to preclude the possibility of detecting it by the ordinary means of physical exploration.

*Prevailing Diseases.*—Dr. Cronyn reports scarlet fever as prevalent; Dr. Samo whooping cough.

Adjourned. J. F. MINER, Sec'y.

